# *POGZ* truncating alleles cause syndromic intellectual disability

**DOI:** 10.1186/s13073-015-0253-0

**Published:** 2016-01-06

**Authors:** Janson White, Christine R. Beck, Tamar Harel, Jennifer E. Posey, Shalini N. Jhangiani, Sha Tang, Kelly D. Farwell, Zöe Powis, Nancy J. Mendelsohn, Janice A. Baker, Lynda Pollack, Kati J. Mason, Klaas J. Wierenga, Daniel K. Arrington, Melissa Hall, Apostolos Psychogios, Laura Fairbrother, Magdalena Walkiewicz, Richard E. Person, Zhiyv Niu, Jing Zhang, Jill A. Rosenfeld, Donna M. Muzny, Christine Eng, Arthur L. Beaudet, James R. Lupski, Eric Boerwinkle, Richard A. Gibbs, Yaping Yang, Fan Xia, V. Reid Sutton

**Affiliations:** Department of Molecular and Human Genetics, Baylor College of Medicine and Texas Children’s Hospital, Houston, TX 77030 USA; Human Genome Sequencing Center, Baylor College of Medicine, Houston, TX 77030 USA; Ambry Genetics, Aliso Viejo, CA 92656 USA; Children’s Hospitals and Clinics of Minnesota, Minneapolis, MN 55102 USA; Arnold Palmer Medical Center, Division of Genetics, Orlando, FL 32806 USA; University of Oklahoma Health Sciences Center, Oklahoma City, OK 73104 USA; Department of Pediatrics, Vanderbilt University Medical Center, Nashville, TN 37212 USA; Exome Laboratory, Baylor Miraca Genetics Laboratory, Houston, TX 77030 USA; Department of Pediatrics, Baylor College of Medicine, Houston, TX 77030 USA; Texas Children’s Hospital, Houston, TX 77030 USA; University of Texas Health Science Center, Houston, TX USA

## Abstract

**Background:**

Large-scale cohort-based whole exome sequencing of individuals with neurodevelopmental disorders (NDDs) has identified numerous novel candidate disease genes; however, detailed phenotypic information is often lacking in such studies. De novo mutations in pogo transposable element with zinc finger domain (*POGZ*) have been identified in six independent and diverse cohorts of individuals with NDDs ranging from autism spectrum disorder to developmental delay.

**Methods:**

Whole exome sequencing was performed on five unrelated individuals. Sanger sequencing was used to validate variants and segregate mutations with the phenotype in available family members.

**Results:**

We identified heterozygous truncating mutations in *POGZ* in five unrelated individuals, which were confirmed to be de novo or not present in available parental samples. Careful review of the phenotypes revealed shared features that included developmental delay, intellectual disability, hypotonia, behavioral abnormalities, and similar facial characteristics. Variable features included short stature, microcephaly, strabismus and hearing loss.

**Conclusions:**

While *POGZ* has been associated with neurodevelopmental disorders in large cohort studies, our data suggest that loss of function variants in *POGZ* lead to an identifiable syndrome of NDD with specific phenotypic traits. This study exemplifies the era of human reverse clinical genomics ushered in by large disease-directed cohort studies; first defining a new syndrome molecularly and, only subsequently, phenotypically.

## Background

Neurodevelopmental disorders (NDDs) reflect a molecularly and phenotypically heterogeneous classification encompassing intellectual disability (ID), microcephaly, and neurobehavioral traits such as autism spectrum disorder (ASD) [1]. Collectively, these traits are quite common, with ASD having a prevalence of ~1 % and ID occurring in ~2–3 % of the population. The delineation of various NDDs is not discrete, as more than one phenotype is often present in an individual. Whole exome sequencing (WES) has revealed that mutations in the same gene may be associated with a broad spectrum of neurobehavioral and neurodevelopmental phenotypes [2, 3].

The clinical and genetic heterogeneity of ASD and other neurobehavioral traits represents a challenge for both molecular diagnoses and new gene discovery [4]. ASD is characterized by impairment in social interaction as well as restricted and stereotyped patterns of interest and activities, and is often accompanied by ID and language delay. Recent advances in personal genome analysis and the assembly of large cohorts for study have begun to identify potentially pathogenic variants in individuals with ASD; however, the majority of ASD loci have yet to be identified and may include gene-regulatory mechanisms such as epigenetic modifications and noncoding RNAs [1]. There is emerging evidence for shared genomic underpinnings and genetic etiologies of neuropsychiatric disorders, including ASD, schizophrenia and ID, as evidenced by both WES studies [5] and the mirror trait manifestations of some copy number variants (CNVs) [6].

The over-representation of de novo mutations in affected individuals illustrates the strong genetic basis for NDDs [5, 7, 8]. The prevalence of de novo CNVs has been extensively explored during the past decade, with a large proportion of ASD cases containing these events [9–12]. Moreover, cognitive phenotypes have been tied to CNV in population cohorts [6, 13]. However, despite the known molecular etiology for ~20–30 % of cases, the genetic etiology in the majority of individuals with ASD is presently unable to be identified.

Genomic approaches to diagnosis include array comparative genomic hybridization (aCGH), whole genome sequencing (WGS) and WES. These approaches have identified the genetic etiology of many diverse Mendelian diseases [14]. Recently, large-scale WES studies have been conducted on a number of NDD cohorts [15–20]. These studies have identified many likely pathogenic single nucleotide variants, resulting in the generation of candidate gene lists for neurodevelopmental traits. However, the large number of individuals in cohort publications precludes the reporting of detailed clinical information for each case and often diagnostically relevant dysmorphology is not recognized until a cohort of individuals with a shared genetic etiology is assembled.

Premature truncating mutations in pogo transposable element with zinc finger domain, or *POGZ* (MIM#614787), have been implicated by WES in three ASD cohort studies [16–18], cohorts examining developmental delay (DD) and ID [15, 16] and one schizophrenia cohort [20]. Additionally, a recently published case report details a missense *POGZ* mutation present in an individual with microcephaly, ASD, ID, and other clinical features [21]. The recurrent identification of *POGZ* in these studies has robustly implicated *POGZ* in diverse NDDs, yet no thorough clinical analysis for *POGZ* variant-associated phenotypic features has been published to date.

Although relatively little is known about the function of POGZ, a recent study detailed the interaction of the zinc finger domain of POGZ with HP1α and the resultant activation of Aurora B kinase [22]. Therefore, POGZ likely plays a role in mitotic progression through heterochromatin formation and chromosomal segregation, and may also influence gene expression. These functions of POGZ are consistent with the presence of deleterious variants in patients with microcephaly and other neurodevelopmental phenotypes [1].

We have identified five *POGZ* disrupting mutations in patients with diverse clinical assessments. These individuals have variable NDDs with shared features comprising ID, global DD, behavioral abnormalities, microcephaly, short stature, strabismus, and shared facial characteristics. Together with eight pathogenic mutations from the literature, we describe the spectrum of phenotypes in individuals with deleterious mutations in *POGZ*. Ascertainment of patients with a shared molecular diagnosis and subsequent phenotypic characterization delineates the clinical presentation common to individuals with *POGZ* mutations. This genotype-driven approach may be especially relevant for the variable presentations of NDDs, and can be used to further clinically characterize candidate genes identified in diverse cohort studies.

## Methods

This study conforms to the Helsinki Declaration and was performed with approval by the Baylor College of Medicine institutional review board, protocol number H-29697, for all sequencing conducted at the Baylor College of Medicine Human Sequencing Center. Written informed consent was obtained from the parents/legal guardians of the patients for publication of this research and any accompanying images. Exome sequence analysis across all sequencing centers followed the guidelines and process for classifying sequencing variants developed by the American College of Medical Genetics and Genomics [23]. Trio-based sequencing allowed for the prioritization of de novo loss-of-function mutations in four patients. An average of 2.5 de novo mutations were identified in each proband, of which *POGZ* was the only truncating allele. Additionally, variants in *POGZ* were selected for confirmation and co-segregation with the phenotype because loss-of-function mutations in this gene have been found in large cohorts of individuals with neuropsychiatric phenotypes. All identified variants in *POGZ* have been deposited into ClinVar under accession numbers SCV000256903, SCV000256904, SCV000256905, SCV000256906, and SCV000256907 in agreement with institutional review board approval and patient consent.

DNA from patient 1 and her mother was subjected to WES at the BCM Human Genome Sequencing Center through the Baylor-Hopkins Center for Mendelian Genomics initiative. Exome sequencing and analysis were performed according to previously described methods [24]. Potential de novo mutations were identified in silico by subtracting variants observed in the mother from those observed in patient 1. Candidate variants were filtered against exome data in publicly available databases, including the 1000 Genomes Project, the National Heart, Lung, and Blood Institute Exome Sequencing Project (ESP), the Atherosclerosis Risk in Communities Study (ARIC) database (http://www2.cscc.unc.edu/aric), and our in-house-generated database of approximately 5000 exomes.

Patients 2 and 3 were identified through diagnostic WES at the Baylor-Miraca Medical Genetics Laboratory. Sequencing and data analysis were conducted as previously described [25]. Briefly, the average coverage for ~20,000 targeted genes (42 Mb of targeted regions, including untranslated exons) was greater than 100×, and more than 95 % of the target bases were covered by at least 20 reads [26].

Patients 4 and 5 were identified via diagnostic exome sequencing at Ambry Genetics (Aliso Viejo, CA, USA). Sequencing and data analysis at Ambry Genetics were conducted as previously described [27]. The mean coverage across the parent–proband trio of patient 4 and patient 5 was greater than 90× with >91 % of the targeted bases covered at >20× .

PCR amplification and Sanger sequencing to verify all candidate mutations were done according to standard procedures and candidate variants were annotated using the *POGZ* RefSeq transcript NM_015100.3. Parental samples, when available, were also tested via standard Sanger sequencing.

## Results

### Clinical reports

Characteristic clinical findings are summarized in Table 1.

**Table 1:** Phenotypic and molecular data

Patient ID	1^a^	2	3	4	5	Patients reported in literature (8)
Genotype	c.2321_2324delCTCT	c.2763dupC	c.833C > G	c.2935C > T	c.2780dupT	
Effect	p.Ser774Cysfs*16	p.Thr922Hisfs*22	p.Ser278*	p.Arg979*	p.Leu927Phefs*17	Frameshift or stopgain (7); missense (1)
Exon number	Exon 15 (CDS 14)	Exon 19 (CDS 18)	Exon 6 (CDS 5)	Exon 19 (CDS 18)	Exon 19 (CDS 18)	
De novo	Not maternal	+	+	+	+	8/8
Age at evaluation	15 years	19 months	3 years 10 months	5 years	4 years 7 months	
Gender	F	F	M	F	F	M (3); F (2); NR (3)
Paternal age	NA	45 years	28 years	NA	30 years	
DD/ID	+	+	+	+	+	+ (7); NR (1)
Behavioral phenotype	+	+	+	NA	+	
ASD	NA	-	+	NA	+	ASD (4); schizophrenia (1); psychiatric abnormality (1)
Hearing loss	-	SNHL	NA	SNHL	SNHL	+ (1); NR (7)
Vision	Mild myopia	Cortical blindness	Astigmatism, hyperopia	Rod-cone dystrophy, anisometropia	Astigmatism	Abnormal ERG, 1; hyperopia and Horner syndrome, 1; optic coloboma, 1
Seizures	-	-	Complex partial seizures	-	-	1 (with hypoglycemia); NR (7)
GI manifestations	NA	+	+	+	+	
Stature <10th percentile	+	+	-	+	+	+ (1); NR (7)
Microcephaly	-	+	-	+	+	+ (2); NR (6)
Brachycephaly	+	+	-	+	+	
Midface hypoplasia	+	-	-	+	+	
Strabismus	-	+	+	+	+	+ (1); NR (7)
Optic nerve hypoplasia	-	-	-	+	+	
Long and flat malar region	+	+	+	+	-	
Flat nasal bridge	+	+	-	-	+	
Broad nasal tip	+	+	+	+	+	
Short philtrum	+	+	+	+	+	
Thin vermillion border	+	+	+	-	+	
Downturned corners of the mouth	+	+	+	+	+	
Palate abnormality	High arched palate	-	Bifid uvula	High arched palate	Cleft palate, high arched palate	
Pointed chin	+	+	-	+	+	
Ears	Over-folded superior helices	Low set, posteriorly rotated	-	Posteriorly rotated	-	+ (1; abnormal outer ear); NR (7)
Micrognathia	-	-	-	-	+ (infancy)	
Prognathism	+	-	-	+	+ (toddler)	
Short neck	+	+	-	-	-	
Brachydactyly	-	-	+	-	+	
Joint laxity	+	+	-	-	-	
Hypotonia	+	+	+	-	+	+ (1); NR (7)
Brain MRI	No structural anomalies	Diffuse T2 hyperintensity, delayed myelination	No structural anomalies	No structural anomalies	Dandy-Walker variant; decreased white matter; enlarged third and fourth ventricles	+1 (thin corpus callosum); NR (7)
Sleep apnea	+	-	-	+	-	
Congenital malformations	-	Congenital diaphragmatic hernia; PDA, PFO/ASD; duplicated renal collecting system	-	-	PFO; left duplicated renal collecting system	

*Abbreviations*: *ASD* atrial septal defect, *GI* gastrointestinal, *F* female, *M* male, *MRI* magnetic resonance imaging, *NA* not available, *NR* not reported, *PDA* patent ductus arteriosus, *PFO* patent foramen ovale, *SNHL* sensorineural hearing loss

^a^Patient 1 was previously reported as patient 2 in Bi et al. [28]

Patient 1 is a 15-year-old female who initially presented in infancy with global DD. She walked at 17 months and her first words were at 18 months. She had a developmental assessment done at 5 years 8 months of age, at which time her motor skills were at age level with a performance IQ of 70, overall IQ of 50, and full scale IQ of 56. Behavioral abnormalities were apparent before 2 years of age, and as she developed, her psychiatric symptoms became more pronounced and included aggressive behavior, self-injury and property destruction that necessitated multiple inpatient psychiatric admissions. Other medical issues included a disturbed sleep cycle that responded to melatonin, mild obstructive sleep apnea, early-onset obesity, and mild myopia. On physical examination at 15 years, her weight was 80.7 kg (97th percentile), height was 152 cm (6th percentile), and head circumference was 52.1 cm (−2 standard deviations). Her body mass index was 34.9 kg/m^2^ (99th percentile). Facial characteristics include brachycephaly, a long and flat malar region, broad and depressed nasal tip, short philtrum, thin vermillion border, downturned corners of the mouth and pointed chin. She had generalized hypotonia with a mildly wide-based and waddling gait. Brain magnetic resonance imaging (MRI), electroencephalogram, and audiology were normal. She was initially suspected to have Smith-Magenis syndrome due to sleep disturbance and global DD but high-density aCGH for chromosome 17p11.2 was normal. Molecular testing revealed a novel missense variant in *RAI1* (NM_030665.3,c.4103A>G, p.S1212G) [28]. This variant was predicted to be benign and polymorphic by PolyPhen-2 and MutationTaster, and is poorly conserved in mammalian evolution. However, the variant was inherited from her father who had a history of learning disability and a personality disorder, and is shared with her sister who had clinical features suggestive of Smith-Magenis syndrome [28]. Thus, we could not conclusively rule out that this missense variant in *RAI1* is contributing to the phenotype present in patient 1, but her more severe developmental disability compared with her father and sister warranted further work-up. Additional diagnostic evaluation was noncontributory and included chromosome analysis, fluorescent in situ hybridization (FISH) for 22q11.2 deletion, *MECP2* sequencing, fragile X testing, lead levels, thyroid function studies, plasma amino acids and urine organic acids. Due to the equivocal interpretation of the *RAI1* variant, research exome sequencing was undertaken.

Patient 2 is a 31-month-old female with microcephaly, short stature, global DD, non-ocular visual impairment, failure to thrive and multiple congenital abnormalities, including diaphragmatic hernia and a duplicated renal collecting system. Initial echocardiogram showed a patent ductus arteriosus and a patent foramen ovale or atrial septal defect, which resolved without surgical intervention. Physical examination revealed brachycephaly, sparse hair, low-set and posteriorly rotated ears, long malar region, hypertelorism with downslanting palpebral fissures, a flat nasal bridge, with a broad and depressed nasal tip, short philtrum, thin vermillion border, downturned corners of the mouth, pointed chin, and a short neck with mild webbing. A full psychological and developmental assessment was performed at two years of age. According to the Mullen Scales of Early Learning her visual reception was within the 20th percentile, fine motor was within the 20th percentile, receptive language was within the 20th percentile, and expressive language was within the 20th percentile. According to the Vineland adaptive behavior scales her communication skills were below the first percentile, daily living skills were below the first percentile, socialization was within the first percentile, motor skills were below the first percentile, and adaptive behavior were below the first percentile. Collectively, the results of her formal assessment indicate significant delays across all areas with relative weakness in expressive language skills. Brain MRI showed nonspecific delayed myelination and diffuse T2 hyperintensity. Skeletal anomalies included multiple Wormian bones and mild hypoplasia of the cervical vertebral bodies. Previous work-up included chromosome analysis and aCGH which were normal as well as very long chain fatty acids. A clinical concern for Pallister-Killian was entertained at birth due to facial features and congenital diaphragmatic hernia.

Patient 3 is a 3-year 10-month-old male with global DD, complex partial seizures and behavioral abnormalities concerning for ASD. He first presented at two months of age due to poor weight gain and poor suck and latch. At 34 months of age, DAYC-2 formal developmental assessment was administered and he scored a General Developmental Index of 67, interpreted as significantly below age-level across all areas. His full scale IQ at 34 months was 71 (third percentile), verbal comprehension was 85 (14th percentile) and non-verbal abilities were 64 (first percentile). Physical examination revealed strabismus, bifid uvula, a wide space between the central incisors, brachydactyly, large thumbs, broad feet, and large toes. Ophthalmology exam at 12 months of age detected hyperopia and astigmatism. Family history was significant for seizures on the maternal side. He has complex partial seizures localizing to the left frontoparietal area. He was initially treated with phenobarbital but switched to levetiracetam and was well-controlled. Due to concern that this drug was contributing to his behavioral issues, the patient was switched to oxcarbazepine. He developed tic-like movements after the second dose and it was stopped. Topiramate was added and he did start having new seizures while he was on both topiramate and levetiracetam but controlled with higher doses of topiramate. Levetiracetam was eventually weaned and the irritability has improved. Previous work-up was unrevealing and included chromosome analysis, aCGH, metabolic testing, Angelman methylation studies and *CREBBP* and *EP300* testing for Rubinstein-Taybi syndrome I and II.

Patient 4 is a 5-year-old female with microcephaly, short stature, global DD, and a history of low muscle tone. Additional medical concerns included feeding difficulties, severe gastroesophageal reflux disease, sensorineural hearing loss, exotropia, and obstructive sleep apnea. Facial characteristics included brachycephaly, a sloping broad forehead, posteriorly rotated ears, upslanting palpebral fissures, long and flat malar region, broad and depressed nasal tip, short philtrum, downturned corners of the mouth, high palate, pointed chin, and relative prognathia. Ophthalmology exam showed exotropia, anisometropia, optic nerve hypoplasia, and rod-cone retinal dystrophy. Brain MRI showed optic nerve hypoplasia with no other structural abnormalities. Skeletal survey was normal other than significant brachycephaly. At age 27 months she underwent a developmental assessment. According to both the Clinical and Auditory Milestones Scale and Cognitive adaptive test she scored at a basal level of 6 months, indicating severe delays across all areas of development with most significant delays to her acquisition of speech and language. Previous molecular testing included aCGH, which revealed a paternally inherited 125-kb copy number gain within chromosome 12q24.12; she had a normal chromosome analysis, an unrevealing targeted microcephaly gene panel, and normal thyroid function studies, plasma amino acids, urine organic acids, and urine mucopolysaccharides.

Patient 5 is a 4-year 7-month-old female with microcephaly, cleft palate, global DD, hypotonia and ASD. She presented at birth with cleft palate, micrognathia and a duplicated renal collecting system. Facial and physical characteristics included broad forehead, brachycephaly, broad and depressed nasal tip, short philtrum, thin vermillion border, downturned corners of the mouth, pointed chin, and small hands with relative brachydactyly. Upon her initial clinical presentation additional features were noted, including auditory neuropathy, immune deficiency, and left duplicated renal collecting system. Ophthalmology exam showed exotropia, astigmatism and small optic nerves. Brain MRI revealed Dandy-Walker variant, decreased white matter, and enlarged third and fourth ventricles. Her original exome report identified compound heterozygous variants of unknown clinical significance in *STIL*, associated with primary microcephaly. Additionally, microarray revealed a paternally inherited Xq21.1 duplication. Previous normal testing included chromosome analysis, FISH for 22q11 deletion, methylation studies for Prader Willi and Angelman syndromes, and *ROR2* sequencing. Other testing, all normal, included urine organic acids, serum amino acids, acylcarnitine, and very long chain fatty acids.

### Phenotypic spectrum

**Fig. 1:**
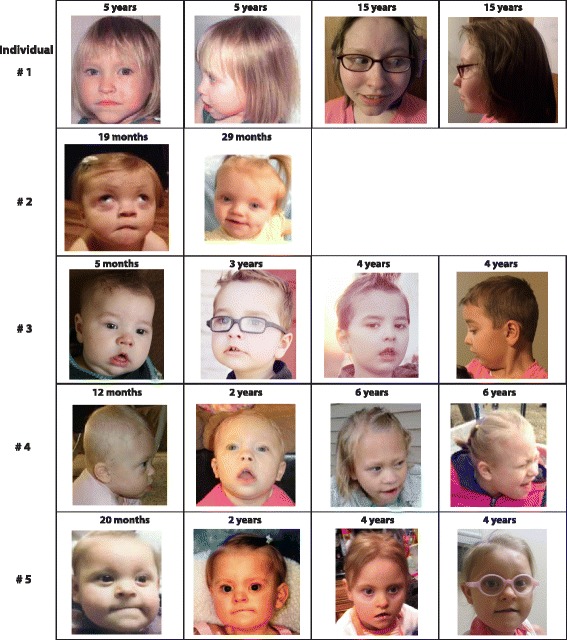
Photographs of patients displaying common facial features. Shared facial dysmorphology among affected individuals includes brachycephaly, long and flat malar region, broad and depressed nasal tip, short philtrum, thin vermillion border, downturned corners of the mouth and pointed chin

The clinical description of the individuals in this study overlaps with the descriptions of previously reported patients [15–20]. All five subjects had global DD and/or ID. Behavioral abnormalities ranged from self-injurious behavior to high-functioning ASD. Additional common features included microcephaly (3/5), stature below the tenth percentile (4/5), hypotonia (4/5), strabismus (4/5), vision abnormalities (5/5), sensorineural hearing loss (3/5), and gastrointestinal manifestations including poor feeding, gastroesophageal reflux, and/or constipation (4/5). Recurrent facial and physical characteristics included hypotonic facies with an open mouth, brachycephaly (4/5), long and flat malar region (4/5), posteriorly rotated ears (2/5), broad nasal tip (5/5), flat nasal bridge (3/5), short philtrum (5/5), thin vermillion border (4/5), downturned corners of the mouth (4/5), high arched palate (3/5), and pointed chin (4/5). An overview of clinical features is presented in Table 1. Pictures of the five patients are shown in Fig. 1.

### Genomic analyses

**Fig. 2:**
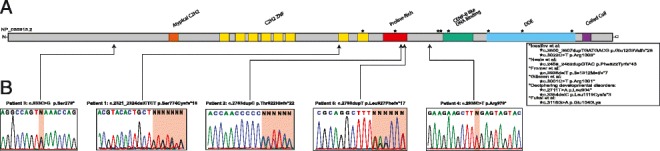
Locations and chromatograms of variants identified. **a** The functional protein domains of *POGZ*. Functional protein domains include zinc-finger domains (*orange* and *yellow*), a predicted proline-rich domain (*red*), CENP-B like DNA binding domain (*green*), DDE transposase domain (*blue*) and a coiled-coil domain (*purple*). The seven truncating mutations and one deleterious missense previously reported are indicated by *stars* above the predicted domains. **b** Truncating mutations identified in the current study are indicated at their respective locations on the protein with *arrows*, and the chromatograms displaying each mutation are presented below the details of the individual nucleotide and amino acid changes

To date, seven de novo mutations leading to premature termination of *POGZ* have been identified in cohorts of individuals with diverse NDDs, including ASD (N = 3), ID (N = 6), DD (N = 2), or schizophrenia (N = 1) [15–20]. The truncating variants result in termination of the protein prior to or within the C-terminal DDE and/or coiled-coil domains. Additionally, a de novo missense mutation in the CENP-B like DNA binding domain of *POGZ* was also reported in a child with ASD, ID and dysmorphic features (Fig. 2) [21].

We identified five patients with heterozygous truncating mutations in *POGZ.* Four of the five variants were confirmed to be de novo in the affected individual; the father of patient 1 was unavailable for analysis but his mother did not harbor the *POGZ* variant. Extensive testing for each of these individuals was negative, with the exception of a paternally inherited missense variant in *RAI1* in patient 1 of uncertain clinical significance [28] and compound heterozygous variants of uncertain clinical significance in *STIL* in patient 5. We identified heterozygous truncating variants in *POGZ* in all five individuals by WES; these five variants were validated by Sanger sequencing (Fig. 2). Publicly available databases, including ARIC and the Exome Aggregation Consortium (ExAC; version 0.3; Cambridge, MA, USA; http://exac.broadinstitute.org) confirmed that the identified variants are unique to our patients. Table 2 provides a molecular description of variants identified in these five patients and eight previously identified cases. Indels resulting in frameshift mutations were present in three of five patients and two of five contained nonsense mutations (one transition and one transversion). Three of the five variants occur in the final exon of *POGZ* and can potentially escape nonsense-mediated decay. However, the two patients with variants predicted to be subject to degradation by nonsense-mediated decay do not have distinguishing phenotypic features from the three terminal exon-containing patients. All five truncating mutations are predicted to result in the loss of greater than 402 amino acids at the C-terminus of POGZ. Loss of this region of the protein is predicted by conceptual translation to result in deletion of the CENP-B like DNA binding domain, the DDE domain and the coiled-coil domain. Thus, the mutations present in each of the five individuals in this study likely result in loss of function. The assertion that heterozygous loss of function mutations in *POGZ* cause diverse NDDs is further supported by the presence of two CNVs encompassing *POGZ* coding regions in the DECIPHER database [29]. The two individuals with small deletions of POGZ are phenotypically described as having DD and additional significant developmental and morphological phenotypes.

**Table 2:** Molecular data for all individuals with variants in *POGZ*

Citation	Patient ID	Gender	Age of evaluation	De novo	Genotype	Protein change	Exon
This study	1	F	1.6 years	Not maternal	c.2321_2324delCTCT	p.Ser774Cysfs*16	15/19
	2	F	1.7 years	+	c.2763dupC	p.Thr922Hisfs*22	19/19
	3	M	3.8 years	+	c.833C > G	p.Ser278*	6/19
	4	F	5.1 years	+	c.2935C > T	p.Arg979*	19/19
	5	F	4 years	+	c.2780dupT	p.Leu927Phefs*17	19/19
Previously reported	Fukai et al. (2015) [21]	M	5 years	+	c.3118G > A	p.E1040K	19/19
	Iossifov et al. (2014) [17]	M	NR	+	c.3022C > T	p.Arg1008*	19/19
	Iossifov et al. (2012) [18]	M	NR	+	c.3600_3607dupTGATGACG	p.Glu1203Valfs*28	19/19
	Neale et al. (2012) [19]	F	NR	+	c.2459_2462dupGTAC	p.Phe822Tyrfs*43	17/19
	Fromer et al. (2014) [20]	F	NR	+	c.3936delT	p.Ile1312Metfs*7	19/19
	Gilissen et al. (2014) [34]	NR	NR	+	c.3001C > T	p.Arg1001*	19/19
	Deciphering Dev. Disorders (2015) [35]	NR	NR	+	c.2711 T > A	p.Leu904*	19/19
		NR	NR	+	c.3354delC	p.Leu1119Cysfs*	19/19

*F* female, *M* male, *NR* not reported

## Discussion

Large cohort studies have associated truncating mutations in *POGZ* with behavioral abnormalities, ID, and DD [16–21]. The identification of numerous candidate genes in these studies highlights their value. However, to thoroughly describe the clinically relevant phenotypes associated with mutation in a given gene, targeted analyses of a molecularly defined cohort are required. Here, we utilized a genotype-first approach to identify shared facial and physical characteristics as well as the spectrum of individual phenotypes resulting from deleterious mutations in *POGZ.*

We identified five patients with novel truncating mutations in *POGZ*, likely resulting in loss-of-function alleles. The description of the five individuals and a review of eight patients from the literature indicate that individuals with mutations in *POGZ* are likely to have global DD, microcephaly, ID, strabismus and variable hearing loss. Comparison of facial and physical characteristics in our five subjects has revealed shared facial features, including brachycephaly, long and flat malar region, broad nasal tip, short philtrum, thin vermillion border, downturned corners of the mouth and pointed chin (Fig. 1). Patients also present with highly variable neurobehavioral phenotypes, ranging from ASD to severe aggressive behaviors similar to what is seen in individuals with Smith-Magenis syndrome (Table 1).

It is possible that patient 1 and patient 2 may have a blended phenotype; patient 1 has a paternally inherited missense mutation in *RAI1*, which is associated with Smith-Magenis syndrome. In addition to the characteristic features, patient 2 also presented with congenital abnormalities, including diaphragmatic hernia, a renal tract anomaly, and heart defects. Other than the de novo *POGZ* mutation, we did not identify additional molecular events that would account for her phenotype. Therefore, although it is possible that *POGZ* truncating mutations could lead to clinical phenotypes including mild congenital abnormalities, an additional unidentified cause in patient 2 could potentially be responsible for her uncharacteristic presentations. Together, our data and a literature review indicate that damaging variants in *POGZ* result in a broad range of features distinguished by global DD, ID, neurobehavioral abnormalities (most often ASD), short stature, hypotonia, strabismus, characteristic facial features, including brachycephaly, long and flat malar region, broad nasal tip, short philtrum, thin vermillion border, downturned corners of the mouth, and pointed chin, and variable hearing loss.

POGZ has not been extensively characterized; the protein product contains eight canonical C2H2-like zinc finger domains that are implicated in protein–protein interactions and have the potential for DNA interactions [30]. The C-terminus of the protein contains a B-like centromere binding domain and a DDE domain (Fig. 2). Interestingly, POGZ interacts with HP1α via the zinc finger domain; this activates Aurora B kinase, indicating a potential role for POGZ in mitotic progression and neuronal differentiation [22]. Similar to *POGZ*, many genes encoding proteins required for proper centrosomal function or chromosome segregation are mutated in patients with microcephaly, demonstrating a common theme of aberrant neuronal proliferation and migration [1, 31].

*POGZ* is expressed in developing cortical projection neurons which have been implicated in a network convergence of neurodevelopmental risk genes [32]. HP1α also strongly interacts with NIPBL, which is mutated in individuals with Cornelia de Lange syndrome. The overlapping molecular networks of POGZ and NIPBL may indicate common mechanisms of transcriptional dysregulation with deleterious mutation of these genes [24].

Although NDDs are typically a clinical diagnosis, molecular genetic testing, including both aCGH and WES, is capable of more precisely defining syndromes and disorders with highly variable phenotypes [33]. With the expanding number of single-gene candidates for NDDs and the variability of these phenotypes, WES was crucial in defining the cohort of five patients presented here. The increasing emergence of candidate genes for various disorders necessitates genotype-driven approaches for describing associated phenotypic spectrums. Furthermore, with a better understanding of the clinical presentations of patients with deleterious *POGZ* mutations, more molecular diagnoses may be forthcoming.

## Conclusions

We report the detailed phenotypic features associated with loss of function variants in *POGZ.* These include a broad range of features distinguished by global DD, microcephaly, ID, neurobehavioral abnormalities (including ASD), short stature, hypotonia, strabismus and variable hearing loss. We also describe characteristic facial features of this disorder that include brachycephaly, long and flat malar region, broad nasal tip, short philtrum, thin vermillion border, downturned corners of the mouth, and pointed chin. Identification of *POGZ* variants and subsequent description of characteristic clinical presentations demonstrate the utility of human reverse genetics in an era of personal genome analyses and clinical genomics.

## Abbreviations

aCGH: Array comparative genomic hybridization
ASD: Autism spectrum disorder
CNV: Copy number variant
DD: Developmental delay
FISH: Fluorescent in situ hybridization
ID: Intellectual disability
MRI: Magnetic resonance imaging
NDD: Neurodevelopmental disorder
WES: Whole-exome sequencing
WGS: Whole-genome sequencing.
